# Molecular mechanisms of *Phytophthora sojae* avirulence effectors escaping host recognition

**DOI:** 10.3389/fmicb.2022.1111774

**Published:** 2023-01-09

**Authors:** Xiaoyuan Hou, Zheng He, Zhengzheng Che, Hengjing Li, Xinwei Tan, Qunqing Wang

**Affiliations:** ^1^Shandong Province Key Laboratory of Agricultural Microbiology, Department of Plant Pathology, College of Plant Protection, Shandong Agricultural University, Tai’an, China; ^2^State Key Laboratory of Crop Biology, Shandong Agricultural University, Tai’an, China

**Keywords:** *Phytophthora sojae*, RXLR effectors, host recognition, plant-microbe interactions, avirulence gene

## Abstract

*Phytophthora sojae* is a well-known destructive oomycete pathogen, which causes soybean stem and root rot and poses a serious threat to global food security. Growing soybean cultivars with the appropriate resistance to *P. sojae* (*Rps*) genes are the primary management strategy to reduce losses. In most *Phytophthora* pathosystems, host resistance protein encoded by a specific *R* gene in the plant recognizes corresponding RxLR effector protein, encoded by an avirulence gene. This gene-for-gene relationship has been exploited to help breeders and agronomists deploy soybean cultivars. To date, 6 *Rps* genes have been incorporated into commercial soybean germplasm and trigger plant immunity in response to 8 *P. sojae* avirulence effectors. The incorporation of Rps genes in the soybean population creates selection pressure in favor of novel pathotypes of *P. sojae*. The 8 avirulence genes evolved to evade the host immune system, driven by genetic selection pressures. Understanding the evading strategies has important reference value for the prevention and control of *Phytophthora* stem and root rot. This investigation primarily highlights the research on the strategies of *P. sojae* avirulence effector evasion of host recognition, looking forward to creating durable resistance genes and thereby enabling successful disease management.

## 1. Introduction

*Phytophthora* stem and root rot (PSRR) caused by *Phytophthora sojae*, is a devastating disease of soybean worldwide, causing US$ 1–2 billion in economic losses worldwide each year (Tyler, 2007). Reducing losses to PSRR primarily relies on growing soybean cultivars with predominant gene(s) that govern resistance to *P. sojae* (*Rps* genes). In PSRR-Soybean pathosystems, specific *Rps* genes that encode nucleotide binding site-leucine rich repeat (NBS-LRR) type of proteins, which recognize effector proteins of the pathogens to induce a defense response (Kang et al., 2012), which is known as the gene-for-gene interaction (Flor and Flor, 1955). Simply put, when *P. sojae* infects a soybean plant, it secretes an effector protein, encoded by an avirulence (*Avr*) gene. If the Rps protein is not contained in the soybean cultivars or the pathogen does not secrete the effector, no recognition is occurring, and disease will develop.

Prior to 2000, *P. sojae* is characterized into 55 races (now known as pathotypes) based upon its virulence on a standard differential set of 8 soybean genotypes that each contain a different *Rps* gene, and a universal susceptible genotype (Grau et al., 2004; Cerritos Garcia et al., 2022). Knowledge of *P. sojae* pathotypes provides important data on the utilization of the resistance genes in a region. In the United States and Canada, *Rps1a*, *Rps1b*, *Rps1c*, *Rps1k*, *Rps3a,* and *Rps6* have been deployed in many commercial soybean cultivars, providing protection to soybean against PSRR in the field promptly (Grau et al., 2004; Dorrance, 2018; Van et al., 2021). Nevertheless, this imposes a directed selection pressure on the pathogen, and the populations of *P. sojae* are becoming more diverse and complex, which evolves to become virulent on the deployed *Rps* genes. For example, a survey conducted in Iowa in 2001–2003 identified 17 pathotypes (Niu, 2004), whereas 37 pathotypes were identified in 2012–2013 (Dorrance et al., 2016). The survey also showed that soybean cultivars with *Rps1a*, *Rps1c*, *Rps1k*, and *Rps3a* genes were mostly overcome by isolates of *P. sojae* in South Dakota (Dorrance et al., 2016). It is interesting that another survey reported a low level of genotypic diversity, and relatively a high level of pathotype diversity was found among the populations between states and within fields (Stewart et al., 2016; Grijalba et al., 2020). That means *P. sojae* avirulence effectors escape from host recognition under a low level of genotypic diversity. It is important to understand the changes in the virulence allele and in turn to help breeders and agronomists develop and deploy soybean cultivars.

So far, 9 *P. sojae* effectors have been shown to be avirulence proteins that were recognized by soybean *Rps* genes *Avr1a* (cognate *Rps* gene: *Rps1a*) (Qutob et al., 2009), *Avr1b* (*Rps1b*) (Shan et al., 2004), *Avr1c* (*Rps1c*) (Na et al., 2014), *Avr1d* (*Rps1d*) (Yin et al., 2013), *Avr1k* (*Rps1k*) (Song et al., 2013), *Avr3a*/*5* (*Rps3a*, *Rps5*) (Qutob et al., 2009), *Avr3b* (*Rps3b*) (Dong et al., 2011a), *Avr3c* (*Rps3c*) (Dong et al., 2009), and *Avr4*/*6* (*Rps4*, *Rps6*) (Dou et al., 2010).

Among of the avirulence effectors shared a conserved RxLR (Arginine-any amino acid-Leucine-Arginine) motif at the N-terminus that mediates effector transport to plant cells (Jiang et al., 2008; Jiang and Tyler, 2012). The RxLR family effectors are unique in oomycetes, including all known avirulence proteins in downy mildews and *Phytophthora* pathogens (Jiang et al., 2008; Chepsergon et al., 2021). The genome of *P. sojae* contains over 400 RxLR family effectors (Tyler et al., 2006). RxLR effectors play an important role in the interaction between *P. sojae* and the host (Wang et al., 2022). The C-terminal of each RxLR effector mediates its specific function (Jiang et al., 2008; He et al., 2019; Zhang P. et al., 2019). Effector proteins are also the potential target for the plant immune system to recognize the invasion. The type of recognition is known as effector-triggered immunity (ETI), a part of the plant immune system (Jones and Dangl, 2006). ETI frequently undergo hypersensitive response (HR) to protect plant healthy from pathogen invasion, forming a “barrier wall” to block pathogen nutrient intake, resulting in rapid local programmed cell death (PCD; Ramírez-Zavaleta et al., 2022). However, pathogens, on the other hand, have developed strategies to suppress or evade ETI (Wang et al., 2011). Due to the frequent emergence of new pathotypes that can overcome resistance genes with time, the durability of a single *Rps* gene is estimated to be 8–20 years (Dorrance et al., 2003; Grau et al., 2004). So far, all of the *Rps genes* for commercial used have been “conquered” in the fields (Tremblay et al., 2021). Therefore, understanding the molecular mechanism of how avirulence effectors evade recognition is an urgent necessity to find out the failure of resistant varieties.

Here, we summarize RxLR avirulence effectors have evolved several distinct mechanisms to evade host recognition, such as by partial or complete deletions, transcriptional variations, transposon insertions, or point mutations. The strategies of evading host recognition reflect the competitive evolution of *P. sojae* and soybean. Both pathogens and their plant hosts continue to compete in this co-evolutionary dynamic.

## 2. Destruction of transcription elements

*PsAvr1b* is the first cloned *Phytophthora* avirulence gene (Shan et al., 2004). Over-expression of *PsAvr1b* made the *P. sojae* transformants more virulent on susceptible soybean, indicating that Avr1b contributes to virulence (Dou et al., 2008). Avr1b interacts with the soybean U-box protein, GmPUB1-1, which is required for some *R* gene mediated resistance (Li et al., 2021). The sequence of the *PsAvr1b* allele from isolate Race2 (P6497), which has a virulent phenotype on *Rps1b* cultivars, has no mutations in the coding region, compared with the allele from avirulent isolate Race1 (P6954) (Shan et al., 2004). However, *PsAvr1b* has a high accumulation of mRNA in the avirulent strains but loses the transcript in Race2 (P6497) (Shan et al., 2004). Research found in virulent strain P6497, enhanced methylation of histone H3 lysine 27 were observed and resulting *PsAvr1b* silenced, allowing the pathogen to evade soybean *Rps1b* resistance gene-mediated defense surveillance (Wang et al., 2020). This indicates the presence of a genetic mechanism, gene silencing due to the destruction of transcription elements, by which avirulence is lost. The naturally occurring *Avr1b*-silenced also suggests that Avr1b is not an essential effectors, and the gene knockout assay also demonstrated that *PsAvr1b* is not required for full virulence of *P. sojae* (Gu et al., 2021).

On the other hand, the transcriptional profiling differences help researcher rapidly identify candidate *Avr* genes, in combination with genetic mapping and whole genome sequence information. Qutob used microarrays to find transcriptional polymorphisms associated with *PsAvr3a* allelic differences (Qutob et al., 2009). *PsAvr3a* is highly expressed in avirulent strains, but it is silenced in virulent strains. The silence is due to an insertion of a transposon-like fragment in its promoter region (Qutob et al., 2009). Promoter and 5′-UTR sequence analysis revealed eight unique mutations in the promoter region of *PsAvr3a*, providing new insights into the escape mechanisms of *P. sojae* (Hu et al., 2021). Further studies revealed that the virulence of *PsAvr3a* is also associated with the accumulation of small RNA. *PsAvr3a* small RNA expression could be detected in the virulent strain ACR10, but not in the avirulent strain Race19 (P7076) (Qutob et al., 2013). The presence of small RNAs generated from the *Avr3a* gene sequence in the parental strain and hybrid offspring was shown to be necessary but not sufficient for gene silencing (Shrestha et al., 2016).

*PsAvr1c* occurs in the *PsAvr1a* gene cluster. Many *P. sojae* strains were found to carry copies of *PsAvr1a* and *PsAvr1c*, but no transcript was detected, indicating that gain of virulence may also result from gene silencing (Na et al., 2014). Also, there are nucleotide substitutions and deletions in the 5′-noncoding region of the gene in the *PsAvr4/6* virulent strain, but no changes in the coding region. Compared to avirulent isolates, virulent isolates Race6 (P7063) and Race30 (PT2004C2.S1) have two nucleotide substitutions (-2C-to-T, -33A-to-G) in the 5′Untranslated Region (UTR), and virulent isolate Race17 (P7074) has one nucleotide substitution (A to G at-33), and deletion of CAGTATCGGG at-170 to -161.The changes make *PsAvr4/6* avoid the recognition of the *Rps4* and *Rps6* (Dou et al., 2010). That indicates the transcriptional profiling differences of *P. sojae Avr* genes represent mechanisms for evasion of *Rps* gene mediated immunity.

It happens that there is a similar case in other oomycetes pathogens, *Phytophthora infestans* Avr2 is also not transcribed in virulent strains, preventing resistance genes recognition (Gilroy et al., 2011; Yang et al., 2020).

## 3. Sequence polymorphism

Four sequenced isolates, Race2 (P6497), Race7 (P7064), Race17 (P7074), and Race19 (P7076), comprise the four major genotypes of *P. sojae* (Tyler et al., 2006; Wang et al., 2011). Of the 378 RxLR effectors predicted to be encoded in four *P. sojae* genomes (Jiang et al., 2008), 147 had small numbers (1–5) of nucleotide substitutions among any of the four *P. sojae* isolates, while 50 genes showed large numbers of substitutions (6 or more; Wang et al., 2011). Avirulence effectors that are encoded by *Avr* genes, often exhibit a high level of polymorphism as a result of *Rps* gene mediated selection.

By the way, *PsAvr1b* has a variety of sequence polymorphisms in virulent strains. For example, there are 21 amino acid mutations in the C-terminal of virulent strain P7076. PsAvr1b^P7076^ successfully evades recognition by resistant proteins due to such drastic sequence mutations (Dou et al., 2008). The substitution of the 174th glycine in *PsAvr3c* by serine greatly reduces its binding affinity to host target Ser/Lys/Arg-rich proteins, thus evading Rps3c-mediated soybean immunity (Dong et al., 2009; Huang et al., 2019).

There are also two distinct amino acid sequences of *PsAvr3b* in the virulent and avirulent strains of *P. sojae*. Compared with the avirulent strain P6497, the virulent strain has two amino acid deletions and 46 amino acid substitutions. The virulent strain encodes the same protein as the P7076 strain, but only 230 amino acids are successfully expressed due to a premature termination codon (Dong et al., 2011a). Through sequence polymorphism, *PsAvr3b* successfully evades recognition of resistance genes, thereby enhancing virulence.

The southern experiment revealed three different patterns of *PsAvr3a*, namely *PsAvr3a-1*, *PsAvr3a-2*, and *PsAvr3a-3*. The two amino acid differences between *PsAvr3a-1* and *PsAvr3a-3*, are derived from avirulent strains. *PsAvr3a-1* copies are also present in the two virulent strains, but no detectable transcripts. Both of them could be recognized by soybeans carrying *Rps3a* and causing cell death. *PsAvr3a-2* is derived solely from virulent strains. Compared with *PsAvr3a-1* and *PsAvr3a-3*, there are a large number of amino acid deletions and mutations, so PsAvr3a-2 evades soybean Rps3a recognition successfully (Qutob et al., 2009; Dong et al., 2011b).

The sequence polymorphism of RxLR effector-encoding genes revealed evidence for positive selection in groups of genes with seven or more mutations, such as in avirulence genes, elicitor genes, and highly expressed effector genes (Wang et al., 2011). *PsAvh238* is a RxLR effector, which was induced during infection nearly 120-fold, could inhibit INF1-triggered PCD but triggers ETI itself. It is reported *PsAvh238* evolved to evade host recognition by mutating a nucleotide site while retaining activity to suppress plant immunity to enhance *P. sojae* virulence. This single-point mutation helped PsAvh238 evade plant recognition (Yang et al., 2017). Plant immunity has also been circumvented by amino acid substitutions in *the Avr3a* allele domains of *P. infestans* (Armstrong et al., 2005; Bos et al., 2006). The avirulence genes *ATR1*, *ATR13*, and *ATR5* of *Peronospora parasitica* also evade the recognition of host disease resistance genes through amino acid sequence variation (Allen et al., 2004; Rehmany et al., 2005; Bailey et al., 2011).

## 4. Gene deletion and copy number variation

The results of virulence test and gene amplification indicated that 11 *P. sojae* strains carry a copy of *Avh6*, which were avirulent in the host carrying *Rps*1d. Five strains lacked *Avh6* and were virulent in the host carrying *Rps*1d.The predicted RxLR effector gene *Avh6* precisely cosegregates with the *Avr1d* phenotype *via* genetic mapping (Na et al., 2013). Avh6 could suppress plant immunity induction by RxLR effectors, including Avh238, and Avh241, and that associated with PiAvr3a and R3a (Wang et al., 2011). The latest report showed that Avr1d physically binds to the E3 ligase GmPUB13, a susceptibility factor, and stabilized GmPUB13 by suppressing the self-ubiquitination (Lin et al., 2021).

Sequencing of *PsAvr1d* genes in 12 *P. sojae* strains revealed two alleles with significant sequence polymorphisms, indicating positive selection is observed for the avirulence gene. Although PsAvr1d undergoes drastic mutations, these mutated sites are not differentially recognized by Rps1d (Yin et al., 2013). By comparing the genomes of avirulent strains and virulent strains, the *PsAvr1d* gene is absent in virulent isolates, the gene deletion formed genotypic diversity and overcome *Rps1d* mediated resistance.

*PsAvr1a* shows copy number variation between strains. There are two copies of *PsAvr1a* in the avirulent strain Race2, while in the virulent strain, such as Race7(P7064), two copies of *PsAvr1a* are deleted, resulting in virulence changes. *PsAvr1a* is also deleted in Race6, Race8, Race9, and Race21, resulting in virulence in soybean carrying *Rps1a* (Qutob et al., 2009).

Recently, the whole genome re-sequencing of *P. sojae* reveals several novel variants that lead to the evading of host resistance, including a complete deletion in *Avr3c* and *Avr1c* (Na et al., 2014; Zhang X. et al., 2019). It is worth mentioning that *Avr3c* family genes are present in some other *Phytophthora* species, and both *PsAvr3c* paralog from *P. sojae* and ProbiAvh89 orthologs from *P. cinnamomi* var. *robiniae* interact with a novel soybean spliceosomal complex protein, GmSKRPs, and reprogram host pre-mRNA alternative splicing to promote infection (Zhang et al., 2018).

A similar situation occurs in other oomycete effectors. *Avr2* deletion was found in some virulent strains of *Phytophthora infestans*, thus avoiding the recognition of resistance gene *R2*(Gilroy et al., 2011).

## 5. Frameshift mutation

The *P.sojae* avirulence gene *PsAvr3b* encodes a secreted NADH and ADP-ribose pyrophosphorylase, and Avr3b-like proteins are conserved in *Phytophthora* species (Dong et al., 2011a). Thus Avr3b might be delivered into plant cells as a Nudix hydrolase to impair host immunity. The *PsAvr3b* gene shows transcriptional polymorphisms between virulent and avirulent strains, indicating that it may try to evade host recognition by decreasing expression levels. However, transcripts of the Avr3b virulence allele remain detectable in both virulent and avirulent strains. By examining *PsAvr3b* polymorphisms among 20 *P. sojae* field isolates from China, 16 avirulent strains encoded a 315 amino acid protein while 4 virulent isolates encoded a protein that is truncated to 230 amino acids by a premature stop codon. The premature termination of the translation resulted in the deletion of about 100 amino acids, which precisely evades avirulence protein recognition (Dong et al., 2011a; Kong et al., 2015).

Rps1k has been the most widely used resistant gene to control PSRR, which responds to two distinct but closely linked Avr genes, *PsAvr1b* and *PsAvr1k* (Song et al., 2013). By sequencing the *PsAvr1k* from 8 isolates, 3 isolates (P6954, P6497, and P7360) contained identical sequences and were avirulent on varieties carrying *Rpslk*. The study also found that the virulent strain has a common 8 nucleotide (TGCTACTT) insertion, leading to an early stop at the front of the region encoding for RxLR motif, resulting in a frameshift and premature termination of translation (Song et al., 2013). The *PsAvr1k* gene shows sequence polymorphisms between virulent strains, indicating that it may try to evade host recognition by nucleotide substitutions, but ultimately chose the strategy of frameshift mutation.

## 6. Cooperative combat

Some effectors, such as PsAvr1d, PsAvr3a, and Avr1k, collaborate to escape the host recognition monitoring, by inhibiting cell death to cover plant immune response induced by other effectors (Wang et al., 2011). To achieve a protective effect, the effector PsAvh73 of *P. sojae* inhibits the death of soybean cells induced by the effector Avr4/6 and enhances virulence (Deb et al., 2018). The ACR9 strain, which expresses *PsAvr1c*, however, is virulent to RpFs1c plants. Maybe because there are other effectors in the strain ACR9 that inhibit ETI caused by Avr1c-Rps1c interaction, thus escaping the monitoring and recognition of plant immunity (Na et al., 2014). *Phytophthora sojae* effector PsCRN127 can inhibit RxLR effector Avh241-induced plant cell death in *N. benthamiana*, as well as improve plant resistance to *Phytophthora* (Zhang et al., 2015).

## 7. Conclusion and future prospects

*Phytophthora sojae* poses a serious threat to global food security, and RxLR-family effectors play an important role in the *P. sojae*-soybean interaction system. A large number of experimental evidence suggests that effectors interfere with host immunity by changing host protein stability, destroying protein complexes, repositioning targets, and other ways, thereby promoting pathogen infection. Simultaneously, the plant immune system also recognizes effectors to trigger plant immunity and inhibit pathogen colonization and expansion (He et al., 2020).

**Figure 1:**
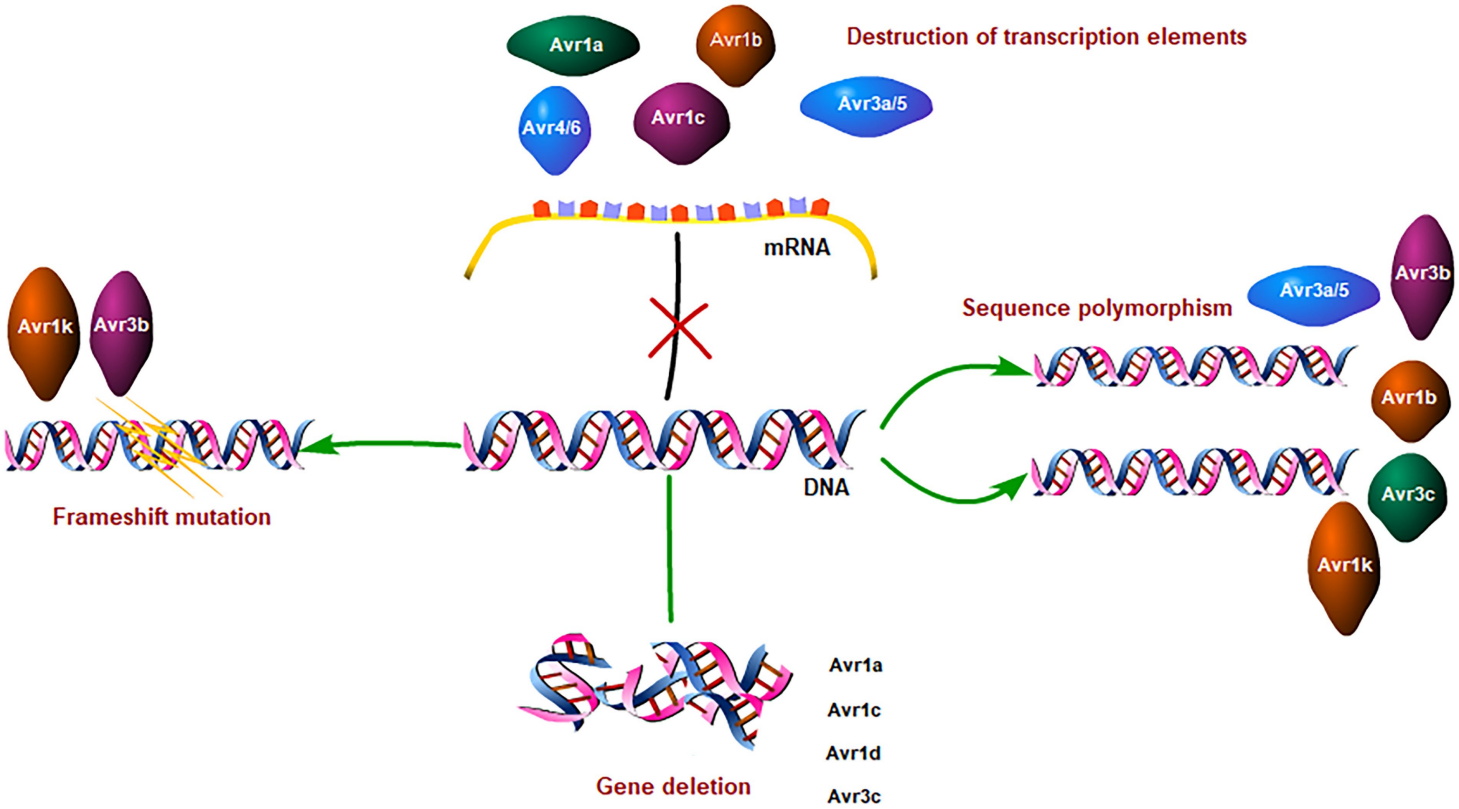
Immune evasion strategies of *Phytophthora sojae* effectors.

Avirulent effectors have evolved a series of strategies to evade the recognition and defense of host plants. They try to evade the surveillance and defense of host plants by the destruction of transcription elements, sequence polymorphisms, gene deletions, frameshift mutations, and cooperative combat (Table 1). As you see, sequence polymorphism is the most common strategy utilized to evade host recognition by these Avr genes. To some extent, the sequence polymorphism reflects the strategies and evolutionary process of *P. sojae* in avoiding recognition by the plant immune system (Ye et al., 2016). Though sometimes nucleotide substitutions were processed, such as Avr1d and Avr1k, the alleles remain avirulence, and thus gene deletion or frameshift mutation happens to help these effectors evade the host recognition. That suggests nucleotide substitutions the first choice for *P.sojae* evolved to evade the host immune system. The research on Avh238 showed that it evades host recognition by mutating a single nucleotide site while retaining activity to suppress plant immunity. That is the biggest challenge to crop breeding (Figure 1).

**Table 1:** Immune evasion strategies of *Phytophthora sojae* Avr genes.

Effectors	Immune evasion strategies	References
*Avr1a*	Destruction of transcription elements, Gene deletion and copy number variation	Qutob et al. (2009) and Na et al. (2014)
*Avr1b*	Destruction of transcription elements, Sequence polymorphism, Methylation	Shan et al. (2004), Dou et al. (2008), Wang et al. (2020), Gu et al. (2021), and Li et al. (2021)
*Avr1c*	Destruction of transcription elements, Gene deletion, Cooperative combat	Na et al. (2014) and Zhang X. et al. (2019)
*Avr1d*	Gene deletion and copy number variation	Yin et al. (2013)
*Avr1k*	Frameshift mutation, Sequence polymorphism	Song et al. (2013)
*Avr3a/5*	Destruction of transcription elements, Sequence polymorphism	Qutob et al. (2009), Qutob et al. (2013), Dong et al. (2011b), Shrestha et al. (2016), and Hu et al. (2021)
*Avr3b*	Sequence polymorphism, Frameshift mutation	Dong et al. (2011a) and Kong et al. (2015)
*Avr3c*	Sequence polymorphism, Gene deletion and copy number variation	Dong et al. (2009), Na et al. (2014), Zhang et al. (2018), Huang et al. (2019), and Zhang X. et al. (2019)
*Avr4/6*	Destruction of transcription elements; Cooperative combat; Sequence polymorphism	Dou et al. (2010), Na et al. (2013), and Deb et al. (2018)

The in-depth analysis of RxLR effector escape mechanisms can provide new insights into the molecular mechanisms underlying the virulence functions of effectors, laying the groundwork for developing novel and practical approaches for sustainable, effective disease prevention and control strategies. Given that many escape mechanisms of *P. sojae* effectors have yet to be investigated, the main challenge will be to develop new system-level methods to identify effector escape strategies, accurately target effectors to reduce virulence and provide new theoretical strategies for improving future soybean disease resistance.

## Author contributions

QW and XH initiated the idea and drafted the manuscript. XH, ZC, and HL performed the literature search and conducted data analysis. ZH and XT critically revised the manuscript. All authors contributed to the article and approved the submitted version.

## Funding

This work was supported by the Outstanding Youth Foundation of Shandong Province (ZR2021YQ20) and the National Natural Science Foundation of China (31972249 and 32172387).

## Conflict of interest

The authors declare that the research was conducted in the absence of any commercial or financial relationships that could be construed as a potential conflict of interest.

## Publisher’s note

All claims expressed in this article are solely those of the authors and do not necessarily represent those of their affiliated organizations, or those of the publisher, the editors and the reviewers. Any product that may be evaluated in this article, or claim that may be made by its manufacturer, is not guaranteed or endorsed by the publisher.
